# Early Diagnosis of Imperforate Hymen in a Pediatric Patient: A Case Report

**DOI:** 10.7759/cureus.83121

**Published:** 2025-04-28

**Authors:** Hamad Alghanim, Khaled Aldraihem, Bandar Alenaze, Yazeed Alrabah, Ahmed Alageel, Abdulaziz Alosaimi, Othman Aldraihem

**Affiliations:** 1 Pediatric Department, King Abdullah Specialized Children’s Hospital, King Abdulaziz Medical City, Ministry of National Guard Health Affairs, Riyadh, SAU; 2 Pediatric Emergency Department, King Abdullah Specialized Children’s Hospital, King Abdulaziz Medical City, Ministry of National Guard Health Affairs, Riyadh, SAU; 3 Pediatric Emergency Department, Children’s Hospital, Taif Health Cluster, Taif, SAU; 4 College of Medicine, King Saud University Medical City, Riyadh, SAU

**Keywords:** cyclical pelvic discomfort, hematocolpos, hematometra, hymen, hymenectomy, hymenotomy, imperforate, urinary retention

## Abstract

An imperforate hymen is a rare congenital anomaly of the female genital tract. It leads to hematocolpos and hematometra due to obstructed menstrual outflow. It can present with primary amenorrhea, cyclic abdominal pain, and urinary retention. It typically remains undiagnosed until puberty. We report the case of an 11-year-old girl who presented with a two-month history of cyclic lower abdominal pain and a two-day history of acute urinary retention and constipation. Physical examination and imaging confirmed the diagnosis of hematometrocolpos secondary to an imperforate hymen. The patient underwent surgical intervention with the successful resolution of symptoms. Timely diagnosis and prompt surgical intervention are crucial to achieve symptom resolution and prevent further complications. Early recognition is essential to avoid a delayed diagnosis that can lead to significant discomfort, urinary retention, and potential reproductive consequences. Proper postsurgical follow-up ensures normal menstrual function, reproductive health, and long-term well-being. This case highlights the importance of early recognition of imperforate hymen to ensure normal reproductive health and prevent long-term complications.

## Introduction

An imperforate hymen is the most common congenital anomaly of the female genital tract and often remains undiagnosed until menarche [1]. It leads to the accumulation of menstrual blood in the vagina and uterus, causing cyclic abdominal pain, urinary retention, and constipation [1]. Its complications include hydronephrosis, infection, and infertility [1]. Early diagnosis and prompt surgical intervention are important to prevent long-term consequences [1]. We report the case of an 11-year-old girl who presented with a two-month history of cyclic lower abdominal pain and a two-day history of acute urinary retention and constipation, who was diagnosed with hematometrocolpos secondary to an imperforate hymen.

## Case presentation

An 11-year-old previously healthy girl presented to the emergency department with a two-month history of cyclic lower abdominal pain. She had developed acute urinary retention and constipation two days before presenting to our emergency department. Examination revealed that the patient experienced visible discomfort. Abdominal examination revealed mild distention; however, the abdomen was soft and non-tender. In-and-out catheterization was performed, which yielded 1500 mL of urine.

**Figure 1:**
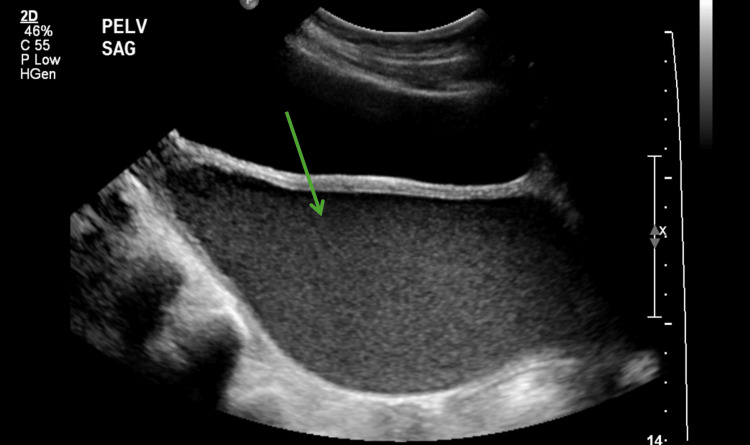
Pelvic ultrasound shows an unremarkable urinary bladder, compressed by the distended vagina, which contains homogeneous low level echoes, likely representing hematometrocolpos (green arrow).

A detailed gynecological examination revealed an imperforate hymen with a bulging bluish membrane. Secondary sexual characteristics, including breast development (Tanner stage III) and pubic hair growth (Tanner stage II), were appropriate for age. Pelvic ultrasonography revealed a normal urinary bladder that was compressed by a distended vagina containing homogeneous low-level echoes, consistent with hematometrocolpos. No internal flow or free fluid was detected. Additionally, left-sided grade 2 hydronephrosis was noted, suggesting significant urinary tract involvement (Figure 1).

The patient was diagnosed with primary amenorrhea secondary to an imperforate hymen complicated by urinary retention and hydronephrosis. She was admitted and underwent a hymenectomy via a cruciate incision that drained approximately 200 mL of blood. Postoperative care included pain management, prophylactic antibiotic administration to prevent infection, and regular urinary function monitoring.

**Figure 2:**
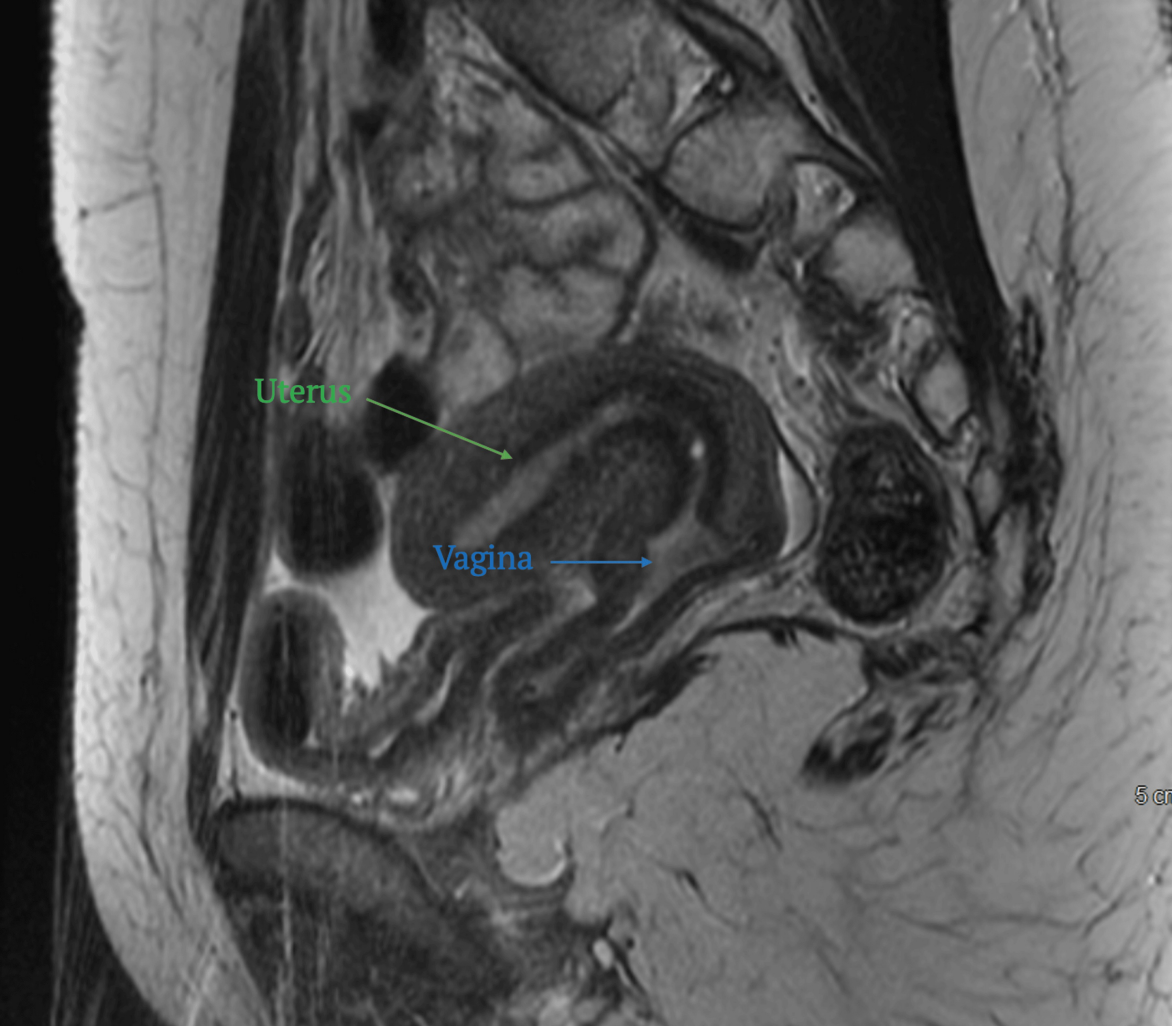
Postoperative MRI revealed reduced distension of the vagina with residual hematocolpos and air-fluid levels. A single, non-septate vagina was observed (blue arrow). The uterus appeared normal, with an arcuate shape and smooth indentation of the fundal endometrial canal measuring 0.5 cm (green arrow).

Postoperative MRI revealed reduced distension of the vagina with residual hematocolpos and air-fluid levels. A single non-septate vagina was observed. The uterus appeared normal, with an arcuate shape and smooth indentation of the fundal endometrial canal measuring 0.5 cm (Figure 2).

The patient recovered uneventfully and was discharged in stable condition. She experienced her first menstrual period 23 days postoperatively. Follow-up confirmed resolution of symptoms, normal menstrual flow, and no further urinary complaints.

## Discussion

Imperforate hymens are often asymptomatic until menarche, when they can cause significant complications such as hematocolpos, hematometrocolpos, and urinary obstruction, with an incidence of 0.05-0.1% [1]. Although imperforate hymen is rare, it remains the most common congenital anomaly of the female genital tract [2]. Imperforate hymen occurs due to the failure of canalization at the most caudal end of the vaginal plate, specifically where it meets the urogenital sinus [3].

The classical presentation includes primary amenorrhea, cyclic pelvic pain, and urinary or bowel symptoms [1]. A systematic review identified 253 cases of imperforate hymen, with the majority presenting with abdominal pain (54.2%), urinary retention (20.3%), and abnormal menstruation (14%) [1]. Early detection of an imperforate hymen is important to prevent complications and avoid unnecessary additional investigations [4]. A significant clinical indicator of a potentially imperforate hymen is a mismatch between a teenager's progressive pubertal development and the absence of menarche [4].

An imperforate hymen is an isolated condition; however, in rare cases, particularly among newborns, maternal estrogen can stimulate fetal secretions, leading to hydrocolpos or hydrometrocolpos, which may present as an abdominal mass in approximately 0.006% of female neonates [5].

An imperforate hymen can occasionally be associated with McKusick-Kaufman syndrome, which involves congenital cardiac abnormalities, polydactyly, and hydrometrocolpos [6,7]. Another syndrome associated with an imperforate hymen is the Bardet-Biedl syndrome, an autosomal recessive disorder characterized by retinal dystrophy or retinitis pigmentosa, postaxial polydactyly, obesity, and nephropathy [8]. Typically, the McKusick-Kaufman syndrome is diagnosed at a very young age, whereas the diagnosis of Bardet-Biedl syndrome is often delayed until teenage years [7].

Familial inheritance of an imperforate hymen has been reported in a few cases; most cases are thought to occur sporadically, and no genetic mutations have been identified [9-12].

An imperforate hymen can be identified by examining the external genitalia, where a bulging bluish hymenal membrane is observed [1]. Abdominal ultrasonography is a reliable and accurate method for detecting pelvic cystic masses [1]. The use of point-of-care ultrasound in emergency departments is encouraged for early diagnosis and prevention of unnecessary workup and imaging [13]. Further MRI is recommended if other anomalies, such as cervical atresia, vaginal atresia, obstructed uterine horn, or transverse or longitudinal vaginal septum, are suspected [14].

The surgical intervention for the treatment of an imperforate hymen should start with the use of a urethral catheter, which should be inserted to precisely identify the urethral location [14]. To prevent urethral injury, the procedure begins with a cruciate or U-shaped incision performed using sharp dissection or needlepoint cautery [14]. Other treatment options have been reported in the literature, including carbon dioxide laser or the insertion of a Foley catheter [15,16]. A systematic review found that surgical intervention, particularly hymenectomy or hymenotomy under general anesthesia, was the primary treatment in 83.5% of the cases, with complications occurring in only 6.6% of the cases [1]. Reported complications included reclosure, vaginal adenosis, vaginal adhesions, urethral sphincter and bladder injury, cicatricial stenosis, amenorrhea, and the need for a second hymenotomy [1].

A novel hymen-sparing surgical technique for treating imperforate hymen using an annular hymenotomy with electrocautery has been reported in the literature [17]. This approach was developed to address sociocultural concerns surrounding virginity preservation. In this study of 15 adolescents, the technique resulted in symptom relief, no complications, and preserved hymenal appearance during follow-up, suggesting it as a safe and culturally sensitive alternative to traditional methods [17].

The use of prophylactic antibiotics in patients with an imperforate hymen is rare. A literature review, which included 253 patients with imperforate hymen, showed that only seven patients received prophylactic antibiotics [1].

## Conclusions

This case underscores the significance of recognizing an imperforate hymen as the cause of primary amenorrhea, cyclic abdominal pain, and urinary retention. Prompt diagnosis and surgical intervention led to successful symptom resolution and the prevention of further complications. Early intervention and follow-up are critical for ensuring normal reproductive health and preventing long-term complications.
